# SARS-CoV-2 variants show resistance to neutralization by many monoclonal and serum-derived polyclonal antibodies

**DOI:** 10.21203/rs.3.rs-228079/v1

**Published:** 2021-02-10

**Authors:** Michael Diamond, Rita Chen, Xuping Xie, James Case, Xianwen Zhang, Laura VanBlargan, Yang Liu, Jianying Liu, John Errico, Emma Winkler, Naveenchandra Suryadevara, Stephen Tahan, Jackson Turner, Wooseob Kim, Aaron Schmitz, Mahima Thapa, David Wang, Andrianus Boon, Dora Pinto, Rachel Presti, Jane O’Halloran, Alfred Kim, Parakkal Deepak, Daved Fremont, Davide Corti, Herbert Virgin, James Crowe, Lindsay Droit, Ali Ellebedy, Pei-Yong Shi, Pavlo Gilchuk

**Affiliations:** Washington University School of Medicine; Washington University School of Medicine; University of Texas Medical Branch; Washington University School of Medicine; UTMB; Washington University; University of Texas Medical Branch; University of Texas Medical Branch; Washington University; Washington University School of Medicine; Vanderbilt University Medical Center; Washington University; Washington University School of Medicine; Washington University School of Medicine; Washington University School of Medicine; Washington University; Washington University in St. Louis; Washington University School of Medicine; Humabs BioMed SA, a subsidiary of Vir Biotechnology, Inc; Washington University School of Medicine; Washington University School of Medicine; Washington University; Washington University; Washington University School of Medicine; VIR; Vir Biotechnology, Washington University School of Medicine; Vanderbilt University Medical Center; Washington University School of Medicine; Washington University School of Medicine; The University of Texas Medical Branch at Galveston; Vanderbilt University Medical Center

**Keywords:** Antibody-based Countermeasures, Vaccine Efficacy, Pseudoviruses, Chimeric Washington Strain, South African Spike Gene, Assay Standardization

## Abstract

Severe acute respiratory syndrome coronavirus 2 (SARS-CoV-2) has caused the global COVID-19 pandemic infecting more than 106 million people and causing 2.3 million deaths. The rapid deployment of antibody-based countermeasures has provided hope for curtailing disease and ending the pandemic^1^. However, the emergence of rapidly-spreading SARS-CoV-2 variants in the United Kingdom (B.1.1.7), South Africa (B.1.351), and elsewhere with mutations in the spike protein has raised concern for escape from neutralizing antibody responses and loss of vaccine efficacy based on preliminary data with pseudoviruses^2–4^. Here, using monoclonal antibodies (mAbs), animal immune sera, human convalescent sera, and human sera from recipients of the Pfizer-BioNTech (BNT162b2) mRNA vaccine, we report the impact on antibody neutralization of a panel of authentic SARS-CoV-2 variants including a B.1.1.7 isolate, a chimeric Washington strain with a South African spike gene (Wash SA-B.1.351), and isogenic recombinant variants with designed mutations or deletions at positions 69–70, 417, 484, 501, and/or 614 of the spike protein. Several highly neutralizing mAbs engaging the receptor binding domain (RBD) or N-terminal domain (NTD) lost inhibitory activity against Wash SA-B.1.351 or recombinant variants with an E484K spike mutation. Most convalescent sera and virtually all mRNA vaccine-induced immune sera tested showed markedly diminished neutralizing activity against the Wash SA-B.1.351 strain or recombinant viruses containing mutations at position 484 and 501. We also noted that cell line selection used for growth of virus stocks or neutralization assays can impact the potency of antibodies against different SARS-CoV-2 variants, which has implications for assay standardization and congruence of results across laboratories. As several antibodies binding specific regions of the RBD and NTD show loss-of-neutralization potency *in vitro* against emerging variants, updated mAb cocktails, targeting of highly conserved regions, enhancement of mAb potency, or adjustments to the spike sequences of vaccines may be needed to prevent loss of protection *in vivo*.

To evaluate the effects of SARS-CoV-2 strain variation on antibody neutralization, we obtained or generated a panel of authentic infectious SARS-CoV-2 strains with sequence variations in the spike gene (Fig 1a–c). A B.1.1.7 isolate had signature changes in the spike gene^5^ including the 69–70 and 144–145 deletions, and N501Y, A570D, D614G, and P681H substitutions. We created a chimeric, fully-infectious SARS-CoV-2 strain with a South African spike gene (Wash SA-B.1.351; D80A, 242–244 deletion, R246I, K417N, E484K, N501Y, D614G, and A701V) and a panel of isogenic spike mutants (D614G, K417N/D614G, E484K/D614G, N501Y/D614G, P681H/D614G, del69–70/N501Y/D614G, E484K/N501Y/D614G, and K417N/E484K/N501Y/D614G) in the Washington strain background (2019n-CoV/USA_WA1/2020 [WA1/2020]). Recombinant viruses and B.1.1.7 were propagated in Vero-TMPRSS2 and Vero-hACE2-TMPRSS2 cells expressing transmembrane protease serine 2 (TMPRSS2) and human angiotensin converting enzyme 2 (hACE2) to prevent the development of adventitious mutations in the spike, especially at or near the furin cleavage site, which accumulate rapidly in Vero E6 cells^6^ and can impact entry pathways and virulence^7^. All viruses were used at low passage (p0 or p1) and deep sequenced to confirm mutations (Supplementary Table S1).

We tested our panel of viruses for antibody-mediated neutralization in Vero-hACE2-TMPRSS2 cells and then repeated some experiments with Vero-TMPRSS2 cells to evaluate for effects of hACE2 over-expression on neutralization^8^. We performed high-throughput focus reduction neutralization tests (FRNTs)^9^ using a panel of neutralizing mAbs recognizing distinct and overlapping epitopes in the RBD including some having potential use in humans. Class 1 antibodies (*e.g*., COV2–2196, COV2–2072, COV2–2050, COV2–2381, COV2–2130, COVOX-384, COVOX-40, 1B07, S2E12, S2H58, and S2X259) are potently neutralizing, block soluble hACE2 binding, and bind multiple proximal sites in the receptor binding motif (RBM) of the RBD as determined by structural or escape mutation analyses (Extended Data Fig 1a)^10–13^; class 2 neutralizing antibodies (*e.g*., S309, SARS2–3, SARS2–10, SARS2–31, SARS2–44) often cross-react with SARS-CoV, bind the base of the RBD (Extended Data Fig 1b), and variably block hACE2 binding (^14^ and L. VanBlargan and M. Diamond, unpublished results); and class 3 neutralizing mAbs (*e.g*., COV2–2676 and COV2–2489) recognize the N-terminal domain (NTD) (Extended Data Fig 1c)^15^.

We performed neutralization tests with the different spike protein variants and the two cell types (Fig 1d–i and Extended Data Fig 2). With the parental WA1/2020 strain, which was derived in Vero CCL-81 cell cultures, neutralization by the majority of class 1 mAbs was similar in Vero-hACE2-TMPRSS2 or Vero-TMPRSS2 cells. In comparison, some class 2 mAbs (*e.g*., S309 and SARS2–44) showed a 4 to 6-fold loss in neutralization potency (EC_50_ value) on Vero-hACE2-TMPRSS2 compared to Vero-TMPRSS2 cells. Moreover, NTD-reactive mAbs (COV2–2489 and COV2–2676) neutralized Vero CCL-81 cell-derived WA1/2020 virus on Vero-hACE2-TMPRSS2 cells but lost activity on Vero-TMPRSS2 cells or when viruses were derived from Vero-hACE2-TMPRSS2 cells (Fig 1f, h–i and Extended Data Fig 2). Given that the expression of hACE2 on recipient Vero cells and the cellular source of virus both can impact the neutralizing activity of mAbs that bind principally outside of the RBM on the spike protein, virus neutralization assays being used for correlation with *in vivo* efficacy of mAbs and vaccines may produce variable results depending on the cell substrate used for virus propagation and infection.

We next assessed the impact of spike protein mutations on mAb neutralization using Vero-hACE2-TMPRSS2 cells (Fig 1h) and Vero-TMPRSS2 cells (Fig 1i). We observed the following patterns with the variant viruses: (a) The D614G or P681H mutations (in the C-terminal region of S1) and the 69–70 deletion (in the NTD) had marginal effects on neutralization potency for the RBM and RBD mAbs we evaluated. It was difficult to assess their impact on the NTD mAbs we tested, since the recombinant viruses were generated in Vero-hACE2-TMPRSS2 cells, and the NTD mAbs neutralized them poorly at baseline; (b) The K417N mutation resulted in ~10-fold reductions in neutralization by mAbs COVOX-40 and SARS2–44 but did not negatively affect other mAbs in our panel. If anything, several class 1 mAbs showed slightly improved inhibitory activity (*P* = 0.002, Wilcoxon matched-pairs signed rank test) with this mutation; (c) Mutation at N501Y reduced the neutralizing activity of COVOX-40 and SARS2–44 slightly but did not alter the potency of other mAbs substantively; this result is consistent with data showing that human convalescent sera efficiently neutralize viruses with N501Y substitutions^16–18^; (d) The E484K mutation negatively impacted the potency of several class 1 antibodies. Compared to the D614G virus, mAbs COV2–2196, COV2–3025, COV2–2381 and S2E12 showed 4- to 5-fold reduced activity against the E484K/D614G virus, and COV2–2050, COVOX-384, 1B07, and S2H58 lost virtually all neutralizing potential; (e) The combination of E484K/N501Y/D614G mutations, which is present in the circulating South African B.1.351 and Brazilian B.1.1.248 strains, showed even greater effects (6- to 13-fold reductions) on the activity of class 1 mAbs COV2–2196, COV2–3025, COV2–2381, and S2E12 mAbs; (f) When we tested class 1 mAbs for inhibition of the Wash SA-B.1.351 virus containing the full South African spike sequence, as expected, several mAbs (COV2–2050, COVOX-384, 1B07, and S2H58) lost activity in both Vero-hACE2-TMPRSS2 and Vero-TMPRSS2 cells. However, the reductions in neutralizing potential by other class 1 mAbs (COV2–2196, COV2–3025, COV2–2381, and S2E12) seen against the E484K/N501Y/D614G mutant virus were absent with Wash SA-B.1.351, which contains additional mutations. The K417N substitution, which is located at the edge of the RBM (Fig 1b) and enhances neutralization by some class 1 mAbs, may compensate for the negative effects on inhibition of the E484K/N501Y mutations. In comparison, we observed a distinct neutralization pattern with Wash SA-B.1.351 for class 2 and 3 mAbs. Because some of these mAbs neutralized Vero-hACE2-TMPRSS2 cell-derived virus poorly when tested in Vero-hACE2-TMPRSS2 cells, we performed parallel experiments in Vero-TMPRSS2 cells. Class 2 mAbs binding the base of the RBD showed small reductions in potency against the Wash SA-B.1.351. However, the two NTD mAbs in class 3 (COV2–2676 and COV2–2489) showed a loss of neutralizing activity against Wash SA-B.1.351 in Vero-TMPRSS2 cells, consistent with recent data with other NTD mAbs using pseudoviruses^4^; (g) With one exception, none of the class 1 mAbs lost activity against the B.1.1.7 isolate on Vero-TMPRSS2 or Vero-hACE2-TMPRSS2 cells. However, we observed moderately diminished neutralizing activity (3- to 11-fold reduction) of some class 2 mAbs (SARS2–31, SARS2–44, and S309) against the B.1.1.7 strain depending on the cell substrate. The reduced potency of S309 mAb against B.1.1.7 strain contrasts with data showing it binds avidly to the B.1.1.7 spike protein on the surface of cells and neutralizes a vesicular stomatitis virus (VSV) pseudotyped with B.1.1.7 spike protein in Vero E6 cells (Extended Data Fig 3a–b). Moreover, one of the NTD class 3 mAbs (COV2–2489) also showed a marked loss of inhibitory activity against the B.1.1.7 strain in both cell types, possibly due to the deletions present in the NTD (69–70 and 144–145)^15^. Together, these data indicate that cell line selection (for both growth of virus stocks and neutralization assays) and hACE2 receptor expression are important variables in assessing the potency of antibodies against different SARS-CoV-2 variants.

Several academic and industry groups have developed mAb cocktails to overcome possible emergence of resistance during therapy^13,19^. We tested two mAb combinations that have potential use in humans (COV2–2196 + COV2–2130 [Vanderbilt University Medical Center; with engineered derivatives being tested in clinical trials by AstraZeneca] and S309 + S2E12 [Vir Biotechnology] for their inhibitory activity against the SARS-CoV-2 variant viruses (Fig 1g–i). The COV2–2196 + COV2–2130 combination generally retained inhibitory activity (< 4-fold reduction) against all strains. Although the S309 + S2E12 combination showed reduced (~10-fold) potency against the E484K/N501Y/D614G strain, it performed effectively against the Wash SA-B.1.351 virus, again suggesting that additional mutations in natural variants (*e.g*., K417N) enable some antibodies to function better against viruses containing E484K and N501Y mutations.

We next assessed how spike protein mutations impacted the neutralizing activity of polyclonal sera obtained from individuals (n = 19) approximately 1 month after mild SARS-CoV-2 infection^20^. Based on experiments with the mAbs, we focused our testing on B.1.1.7, Wash SA-B.1.351, and WA1/2020 with mutations at D614G, K417N/D614G, E484K/N501Y/D614G, or K417N/E484K/N501Y/D614G, and used Vero-hACE2-TMPRSS2 cells for virus propagation and neutralization assays (Fig 2 and Extended Data Fig 4). These results were compared to data with similarly passaged WA1/2020 D614G and revealed the following: (a) significant differences in neutralization were not observed with the K417N/D614G or B.1.1.7 strains (Fig 2a–b), both of which lack the E484K mutation; (b) serum neutralization titers were lower against E484K/N501Y/D614G (5-fold, *P* < 0.0001), K417N/E484K/N501Y/D614G (3.5-fold, *P* < 0.0001), and Wash SA-B.1.351 (4.5-fold, < 0.0001) viruses (Fig 2c–e), all of which contain the E484K mutation. A heatmap analysis showed that most individuals lost neutralizing activity against all three viruses containing the E484K and N501Y mutations (Fig 2f).

Given that viruses containing changes at positions 484 and 501 escape neutralization by serum from convalescent humans, we next examined the effects of vaccine-induced antibody responses. Initially, we interrogated sera from mice (n = 10), hamsters (n = 8), and non-human primates (NHP [rhesus macaques], n = 6) obtained one month after immunization with ChAd-SARS-CoV-2, a chimpanzee adenoviral vectored vaccine encoding for a prefusion stabilized form of the spike protein^21–23^. We assessed serum neutralization of B.1.1.7, Wash SA-B.1.351, and recombinant WA1/2020 with mutations at D614G, K417N/D614G, E484K/N501Y/D614G, or K417N/E484K/N501Y/D614G using virus derived from and tested on Vero-hACE2-TMPRSS2 cells (Extended Data Fig 5). For serum samples from mice, when comparing the GMTs of neutralization to the WA1/2020 D614G strain, we observed a slight increase (1.9-fold, *P* < 0.05) with K417N/D614G (Fig 3b), decreases with E484K/N501Y/D614G (9-fold, *P* < 0.001; Fig 3c), K417N/E484K/N501Y/D614G (5-fold, *P* < 0.01; Fig 3d), and Wash SA-B.1.351 (5-fold, *P* < 0.01; Fig 3e), yet no significant differences with B.1.1.7. (Fig 3a). In a heatmap plot (Fig 3p), 9 of the 10 mouse sera show a loss of neutralizing activity against multiple viruses containing the E484K mutation. In hamsters, the results were similar. We observed a marked decrease (10- to 12-fold, *P* < 0.01) in serum neutralization of E484K/N501Y/D614G, K417N/E484K/N501Y/D614G, and Wash SA-B.1.351 (Fig 3h–j). Statistically significant differences in neutralization were not observed with K417N/D614G and B.1.1.7 (Fig 3f, g). This pattern was reflected at the individual sample level (Fig 3q). In NHPs, we also observed a substantial decrease (9- to 11-fold, *P* < 0.05) in serum neutralization of E484K/N501Y/D614G, K417N/E484K/N501Y/D614G, and Wash SA-B.1.351 (Fig 3m–o), but no significant difference in inhibition with K417N/D614G or B.1.1.7 (Fig 3k–l). The heatmap analysis showed that all NHP sera consistently exhibited reduced neutralizing activity against viruses containing the E484K mutation (Fig 3r).

Because samples from human immunization trials with ChAd-SARS-CoV-2 are not yet available, we interrogated sera from 24 individuals who received the Pfizer-BioNTech (BNT162b2) vaccine, a lipid nanoparticle encapsulated-mRNA that encodes a similar membrane-bound, prefusion stabilized form of the full-length SARS-CoV-2 spike protein^24^. We tested sera (Extended Data Fig 6 and 7) for neutralization of our panel of SARS-CoV-2 variants (Fig 4a–e). Compared to the WA1/2020 D614G cloned variant, we observed moderate reductions in neutralizing activity (GMTs) of B.1.1.7 (2-fold, *P* < 0.01; Fig 4a) and E484K/N501Y/D614G (4-fold, *P* < 0.0001; Fig 4c) and larger decreases in activity against Wash SA-B.1.351 (10-fold, *P* < 0.0001; Fig 4d), with all subjects showing substantially reduced potency (Fig 4e), results that agree with pseudovirus studies^4^. Significant differences in neutralizing activity were not detected with K417N/D614G (Fig 4b).

We also evaluated the impact of cell substrate and hACE2 receptor expression on neutralizing activity of serum samples from convalescent adults enrolled at approximately one month after infection (Fig 5a–c) and from BNT162b2 mRNA-vaccinated individuals (Fig 5d–f). Given the limited remaining serum quantities, we performed neutralization experiments only with WA1/2020, B.1.1.7, and Wash SA-B.1.351 viruses using Vero-TMPRSS2 cells. Using next-generation sequence analysis of viral genomes, we confirmed additional mutations did not occur during passage in Vero-TMPRSS2 cells (Supplementary Table S1). These experiments (Extended Data Fig 8) revealed the following: (a) Convalescent and vaccine sera showed reductions in neutralizing activity of B.1.1.7 compared to the WA1/2020 virus. Whereas sera from the vaccinated individuals inhibited B.1.1.7 virus infection less efficiently (3.6-fold, *P* < 0.01; Fig 5d), convalescent sera showed a trend towards reduced neutralization that did not attain statistical significance (2.4-fold, P = 0.08; Fig 5a). (b) In comparison, sera from both convalescent and vaccinated individuals showed a marked 10- to 13-fold reduction (*P* < 0.01) in neutralizing potency against the Wash SA-B.1.351 virus (Fig 5b, e). The results for the vaccine sera were similar in magnitude between Vero-hACE2-TMPRSS2 and Vero-TMPRSS2 cells (see also Fig 4a, d) and suggest that cellular expression of hACE2 does not markedly impact functional outcome. However, we generally observed greater decreases in neutralizing activity against the B.1.1.7 strain with convalescent sera in Vero-TMPRSS2 cells than in Vero-hACE2-TMPRSS2 cells (see Fig 2a), possibly because NTD antibodies are produced at higher levels during natural infection than vaccination and lose binding to B.1.1.7 viruses because of deletions in the NTD^25^.

Our *in vitro* experiments using a B.1.1.7 isolate and engineered variants in the backbone of the WA1/2020 strain establish that mutations in the spike can impact the potency of antibody neutralization. Some neutralizing mAbs targeting the base of the RBD or NTD showed reduced activity against the B.1.1.7 isolate, whereas others targeting the RBM or NTD failed to inhibit infection of Wash SA-B.1.351 or variants containing the E484K mutation. These finding are potentially important because the RBM has functional plasticity^26,27^, and additional mutations in this region that occur as the pandemic evolves could further impact the efficacy of mAb therapies or vaccines. Our results establishing the E484K substitution as a vulnerability for multiple neutralizing mAbs are consistent with deep mutational scanning or VSV-SARS-CoV-2-based neutralization escape screening campaigns^26,28,29^. However, several other highly neutralizing mAbs (*e.g*., COV2–2196, COV2–2381, COV2–3025, and S2E12) showed intact or only slightly diminished inhibitory activity against the suite of variant viruses we tested. Moreover, cocktails of mAbs binding different epitopes in spike protein overcame virus resistance to individual mAbs. Alternative approaches to addressing the diminished mAb neutralization activity by variant SARS-CoV-2 lineages include targeting of conserved regions of the spike and identifying clonal mAb variants with greater potency, such that a given dose of mAb can protect against a range of variants despite some decrease in neutralization activity.

Our studies with human sera from convalescent subjects and recipients of the BNT162b2 mRNA vaccine, and animal sera after immunization with a vaccine encoding a similar spike gene, demonstrate a lower potency of neutralization against E484K and N501Y-containing viruses (note: we did not perform studies with the single-mutation viruses, due to limited serum availability). This observation is unexpected given that antibody responses in animals and humans are polyclonal and in theory, should overcome resistance associated with individual mutations and loss of activity of particular B cell clones.

Our analyses agree with some studies showing substantial or complete escape against spike proteins corresponding to the South African lineage (B.1.351 or 501Y.V2) by antibodies in convalescent or vaccine-immune plasma using lentiviral-based pseudotype neutralization assays^2,3,17^. Moreover, they are consistent with studies showing loss of neutralization potency of human convalescent serum against VSV-SARS-CoV-2 chimeric virus variants containing the E484K mutation^30^ and selection of escape E484K mutants under serial passage of convalescent COVID-19 plasma^31^. These findings may have therapeutic implications, as immune plasma derived from individuals infected early during the pandemic might fail to protect patients infected with more recent isolates containing the E484K mutation.

Our studies focused exclusively on the impact of sequence changes in the spike protein on antibody neutralization in cell culture. Despite observing marked differences in serum neutralizing activity against authentic SARS-CoV-2 variant viruses, it remains unclear how this finding translates into effects on protection in the context of secondary infection or infection after vaccination with platforms using historical spike gene sequences. Although serum neutralizing titers are an anticipated correlate of protection^32^, this measurement does not account for Fc effector functions; Fcg receptor or complement protein engagement by non-, weakly-, or strongly- neutralizing antibodies that bind the SARS-CoV-2 spike protein on the surface of infected cells could confer substantial protection^33–35^. Also, the role of memory T or B cells in protection against variant viruses is unknown and could prevent severe infection even in the setting of compromised serum antibody responses^36–38^.

Moreover, the field still does not know whether Vero or other cell-based neutralization assays predict antibody-mediated protection. Indeed, primary cells targeted by SARS-CoV-2 *in vivo* can express unique sets of attachment and entry factors^39^, which could impact receptor and entry blockade by specific antibodies. We observed that cell line used for growth of viral stocks or neutralization assays affects the potency of monoclonal or serum-derived antibodies against different SARS-CoV-2 variants. Such results may impact the congruity of data across laboratories and interpretation of effects of viral variants on vaccine efficacy. As an example, a recent study with Vero E6 cell-derived SARS-CoV-2 with a spike protein containing some of the South African mutations (E484K, N501Y, and D614G) showed only a small 1.2-fold decrease in neutralization potency by BNT162b2 mRNA vaccine-elicited human sera^16^. When we compared neutralization of deep-sequenced confirmed p0 (Vero E6 cell-produced) and p1 (Vero-hACE2-TMPRSS2 cell-produced) K417N/E484K/N501Y/D614G viruses by immune serum from vaccinated mice, hamsters, or NHPs, or naturally infected humans in recipient Vero-hACE2-TMPRSS2 cells, the p0 viruses produced in Vero E6 cells were neutralized more efficiently (~3-fold, *P* < 0.05) than the p1 viruses produced in Vero-hACE2-TMPRSS2 cells (Extended Data Fig 9). We speculate that TMPRSS2 might modify the spike protein of authentic SARS-CoV-2 in the producer cell such that some classes of antibodies no longer efficiently block infection of the recipient cell.

While our analysis of neutralizing antibody responses with authentic infectious SARS-CoV-2 variants on Vero-hACE2-TMPRSS2 and Vero-TMPRSS2 cells suggests that adjustments to some therapeutic antibody cocktails or existing spike sequences in vaccines might be necessary, corroborating *in vivo* studies are needed. Sequential infection and/or vaccination/infection studies in animals and analysis of vaccine efficacy in the setting of new variant infections ultimately will determine the impact of emerging SARS-CoV-2 lineages, especially those containing E484K mutations.

## Methods

### Cells.

Vero E6 (CRL-1586, American Type Culture Collection (ATCC), Vero-TMPRSS2, and Vero-hACE2-TMPRSS2 cells were cultured at 37°C in Dulbecco’s Modified Eagle medium (DMEM) supplemented with 10% fetal bovine serum (FBS), 10 mM HEPES pH 7.3, 1 mM sodium pyruvate, 1× non-essential amino acids, and 100 U/ml of penicillin–streptomycin. Vero-TMPRSS2 were generated after lentivirus transduction. Briefly, human TMPRSS2 was cloned into a pLX304 lentiviral vector (gift of S. Ding, Washington University) with a C-terminal V5 tag and a blasticidin selection marker. TMPRSS2-V5-encoding vectors were packaged as lentiviruses and Vero E6 cells were transduced. Vero E6 cells stably expressing TMPRSS2 were selected under blasticidin (5 mg/mL), and surface TMPRSS2 expression was confirmed using an anti-V5 antibody (Thermo Fisher 2F11F7) or anti-TMPRSS2 mAb (Abnova, Clone 2F4) and APC-conjugated goat anti-mouse IgG (BioLegend, 405308). Vero-hACE2-TMPRSS2 were obtained as a generous gift (A. Creanga and B. Graham, NIH).

### Viruses.

The 2019n-CoV/USA_WA1/2020 isolate of SARS-CoV-2 was obtained from the US Centers for Disease Control (CDC). The B.1.1.7 isolate was obtained from an infected individual. Individual point mutations in the spike gene (D614G, K417N/D614G, E484K/D614G, N501Y/D614G, P681H/D614G, del69–70/N501Y/D614G, and E484K/N501Y/D614G) were introduced into an infectious cDNA clone of the 2019n-CoV/USA_WA1/2020 (WA1/2020) strain as described previously^41^. Nucleotide substitutions were introduced into a subclone puc57-CoV-2-F5–7 containing the spike gene of the SARS-CoV-2 wild-type infectious clone^42^. The South African variant spike gene (B.1.351) was produced synthetically by Gibson assembly. The full-length infectious cDNA clones of the variant SARS-CoV-2 viruses were assembled by *in vitro* ligation of seven contiguous cDNA fragments following the previously described protocol^42^. *In vitro* transcription was then performed to synthesize full-length genomic RNA. To recover the mutant viruses, the RNA transcripts were electroporated into Vero E6 cells. The viruses from the supernatant of cells were collected 40-h later and served as p0 stocks^43^. All viruses were passaged once in Vero-hACE2-TMPRSS2 or Vero-TMPRSS2 cells and subjected to deep sequencing after RNA extraction to confirm the introduction and stability of substitutions (Supplementary Table 1). Viral RNA from cell culture supernatants was used to generate next generation sequencing libraries using either the Illumina TruSeq Stranded Total RNA Library Prep with Ribo-Zero kit or the Illumina Stranded Total RNA Prep, Ligation with Ribo-Zero Plus kit per the manufacturer’s protocol. The final indexed libraries were quantified using Agilent’s Bioanalyzer and pooled at an equal molar concentration. Illumina’s NextSeq sequencer was used to generate paired end 150 base pair reads. Raw sequencing data was processed using fastp^44^ v.0.20.1 (https://github.com/OpenGene/fastp) to trim adapters and filter out sequence with < Q30. Alignment to the SARS-CoV-2 reference genome (MN908947.3) was performed using BWA^45^ v0.7.17-r1188 (http://bio-bwa.sourceforge.net). DeepVariant^46^ v1.1.0 (https://github.com/google/deepvariant) was used to call variants with an allele frequency >= 50%. Variants were annotated using SNPEff^47^ 5.0c (https://sourceforge.net/projects/snpeff/). All virus preparation and experiments were performed in an approved Biosafety level 3 (BSL-3) facility.

### Monoclonal antibodies.

The human mAbs studied in this paper (COV2–2196, COV2–2072, COV2–2050, COV2–2381, COV2–2130, COVOX-384, COVOX-40, S309, S2E12, S2H58, S2X333, VIR-7381, and S2X259) were isolated from blood samples from individuals in North America or Europe with previous laboratory-confirmed symptomatic SARS-CoV or SARS-CoV-2 infection. The original clinical studies to obtain specimens after written informed consent were previously described^10,13,14,25^ and approved by the Institutional Review Board of Vanderbilt University Medical Center, the Institutional Review Board of the University of Washington, the Research Ethics Board of the University of Toronto, and the Canton Ticino Ethics Committee (Switzerland). Chimeric mAb 1B07 with a murine Fv and human Fc (human IgG1) were isolated from C57BL/6 mice immunized with recombinant spike and RBD proteins and described previously^12^. Murine mAbs were generated in BALB/c or C57BL/6 mice immunized with recombinant spike and RBD proteins and described previously^28,30^.

### Human immune sera.

Multiple sources of human serum samples were used in this study: Convalescent serum samples were obtained from a cohort recruited from the St. Louis metropolitan area who experienced mild SARS-CoV-2 infection. None of those patients required intubation, and the study was approved by Washington University School of Medicine Institutional Review Board (202003186 (WU353)). The serum samples from individuals immunized with the Pfizer-BioNTech (BNT162b2) mRNA vaccine were obtained prior to primary immunization or one week after boosting from young adults, and the studies were approved by Washington University School of Medicine Institutional Review Board (202012081 (WU368) and 202012084 (COVaRiPAD))

### Mouse, hamster, and NHP immune sera.

The mouse, hamster, and NHP immune sera were obtained one month after intranasal immunization with ChAd-SARS-CoV-2, a chimpanzee adenoviral vectored vaccine encoding for a prefusion stabilized form of the spike protein. Details of the immunization protocol and functional analyses have been described elsewhere^21–23^.

### Focus reduction neutralization test.

Serial dilutions of mAbs or serum were incubated with 10^2^ focus-forming units (FFU) of different strains or variants of SARS-CoV-2 for 1 h at 37°C. Antibody-virus complexes were added to Vero-hACE2-TMPRSS2 or Vero-TMPRSS2 cell monolayers in 96-well plates and incubated at 37°C for 1 h. Subsequently, cells were overlaid with 1% (w/v) methylcellulose in MEM supplemented with 2% FBS. Plates were harvested 24 h later by removing overlays and fixed with 4% PFA in PBS for 20 min at room temperature. Plates were washed and sequentially incubated with an oligoclonal pool of SARS2–2, SARS2–11, SARS2–16, SARS2–31, SARS2–38, SARS2–57, and SARS2–71 anti-S antibodies and HRP-conjugated goat anti-mouse IgG in PBS supplemented with 0.1% saponin and 0.1% bovine serum albumin. SARS-CoV-2-infected cell foci were visualized using TrueBlue peroxidase substrate (KPL) and quantitated on an ImmunoSpot microanalyzer (Cellular Technologies).

### ELISA.

Assays were performed in 96-well plates (MaxiSorp; Thermo) coated with 100 μL of recombinant spike or RBD protein^12^ in PBS, and plates were incubated at 4 °C overnight. Plates were then blocked with 10% FBS and 0.05% Tween20 in PBS. Serum were serially diluted in blocking buffer and added to the plates. Plates were incubated for 90 min at room temperature and then washed 3 times with 0.05% Tween-20 in PBS. Goat anti-human IgG-HRP (Jackson ImmunoResearch, 1:2,500) was diluted in blocking buffer before adding to wells and incubating for 60 min at room temperature. Plates were washed 3 times with 0.05% Tween-20 in PBS, and then washed 3 times with PBS before the addition of peroxidase substrate (SigmaFAST o-Phenylenediamine dihydrochloride, Sigma-Aldrich). Reactions were stopped by the addition of 1 M HCl. Optical density measurements were taken at 490 nm. The half-maximal binding dilution for each serum or plasma sample was calculated using nonlinear regression. The limit of detection was defined as 1:30.

### Transient expression of recombinant SARS-CoV-2 spike proteins and flow cytometry.

The full-length S gene of SARS-CoV-2 strain (SARS-CoV-2-S) isolate BetaCoV/Wuhan-Hu-1/2019 (accession number MN908947) carrying D614G was codon-optimized for expression in hamster cells and cloned into the pcDNA3 expression vector. Amino acid substitutions for B.1.1.7, P.1 (Brazilian lineage: L18F, T20N, P26S, D138Y, R190S, K417T, E484K, N501Y, H655Y, T1027I, and V1167F), and B.1.351 variants were introduced by overlap extension PCR. Briefly, DNA fragments with overlap sequences were amplified by PCR (step 1). Mutations were introduced by amplification with primers with similar melting points (Tm). Deletion of the C-terminal 21 amino acids was introduced to increase surface expression of the recombinant spike. Next, three contiguous overlapping fragments were fused by a first overlap PCR (step 2) using the utmost external primers of each set, resulting in three larger fragments with overlapping sequences. A final overlap PCR (step 3) was performed on the three large fragments using the utmost external primers to amplify the S gene and the flanking sequences including the restriction sites KpnI and NotI. This fragment was digested and cloned into the expression plasmid phCMV1. For all PCR reactions, the Q5 Hot Start High fidelity DNA polymerase was used (New England Biolabs), according to the manufacturer’s instructions and adapting the elongation time to the size of the amplicon. After each PCR step, the amplified regions were separated on agarose gel and purified using Illustra GFX™ PCR DNA and Gel Band Purification Kit (Merck KGaA).

Expi-CHO cells were transiently transfected with SARS-CoV-2-S expression vectors using Expifectamine CHO Enhancer. Two days later, cells were collected for immunostaining with mAbs. An Alexa647-labelled secondary antibody anti-human IgG Fc was used for detection. Binding of mAbs to transfected cells was analyzed by flow-cytometry using a ZE5 Cell Analyzer (Biorard) and FlowJo software (TreeStar). Positive binding was defined by differential staining of CoV-S-transfectants versus mock-transfectants.

### SARS-CoV-2 pseudotyped virus production.

293T/17 cells were seeded in 10-cm dishes for 80% next day confluency. The next day, cells were transfected with the plasmid pcDNA3.1(+)-spike-D19 (encoding the SARS-CoV-2 spike protein) or pcDNA3.1(+)-spike-D19 variants using the transfection reagent TransIT-Lenti according to the manufacturer’s instructions. One day post-transfection, cells were infected with VSV-luc(VSV-G) at an MOI of 3. The cell supernatant containing SARS-CoV-2 pseudotyped virus was collected at day 2 post-transfection, centrifuged at 1,000 x *g* for 5 min to remove cellular debris, aliquoted and frozen at −80°C. The SARS-CoV-2 pseudotyped virus preparation was quantified using Vero E6 cells seeded at 20,000 cells/well in clear bottom black 96 well plates the previous day. Cells were inoculated with 1:10 dilution series of pseudotyped virus in 50 μL DMEM for 1 h at 37°C. An additional 50 μL of DMEM was added, cells were incubated overnight at 37°C. Luciferase activity was quantified with Bio-Glo reagent by adding 100 μL of Bio-Glo (diluted 1:1 in PBS), incubated at room temperature for 5 min, and relative light units (RLU) were read on an EnSight or EnVision plate reader.

### Neutralization of SARS-CoV-2 pseudotyped virus.

Vero E6 cells were seeded into clear bottom black-walled 96-well plates at 20,000 cells/well in 100 μL medium and cultured overnight at 37°C. Twenty-four hours later, 1:3 8-point serial dilutions of mAb were prepared in medium, with each dilution tested in duplicate on each plate (range: 10 μg/mL to 4 ng/mL final concentration). Pseudovirus was diluted 1:25 in medium and added 1:1 to 110 μL of each antibody dilution. Pseudovirus:antibody mixtures were incubated for 1 h at 37°C. Media was removed from the Vero E6 cells and 50 μL of pseudovirus:antibody mixtures were added to the cells. One hour post-infection, 100 μL of medium was added to wells containing pseudovirus:antibody mixtures and incubated for 17 h at 37°C. Media then was removed and 100 μL of Bio-Glo reagent (diluted 1:1 in DPBS) was added to each well. The plate was shaken on a plate shaker at 300 RPM at room temperature for 20 min, and RLUs were read on an EnSight or EnVision plate reader.

### Data availability.

All data supporting the findings of this study are available within the paper and are available from the corresponding author upon request. Deep sequencing datasets of viral stocks are available at NCBI BioProject PRJNA698378 (https://dataview.ncbi.nlm.nih.gov/object/PRJNA698378?reviewer=g0mic4v5t4e1tpssk63p990suu).

### Statistical analysis.

All statistical tests were performed as described in the indicated figure legends. Non-linear regression curve fitting was performed to calculate EC_50_ values. Statistical significance was calculated using a non-parametric Wilcoxon matched-pairs signed rank test and Prism 8.0. The number of independent experiments used are indicated in the relevant Figure legends.

## Supplementary Material

Supplement

Supplement

Supplement

Supplement

Supplement

Supplement

Supplement

Supplement

Supplement

Supplement

## Figures and Tables

**Figure 1:**
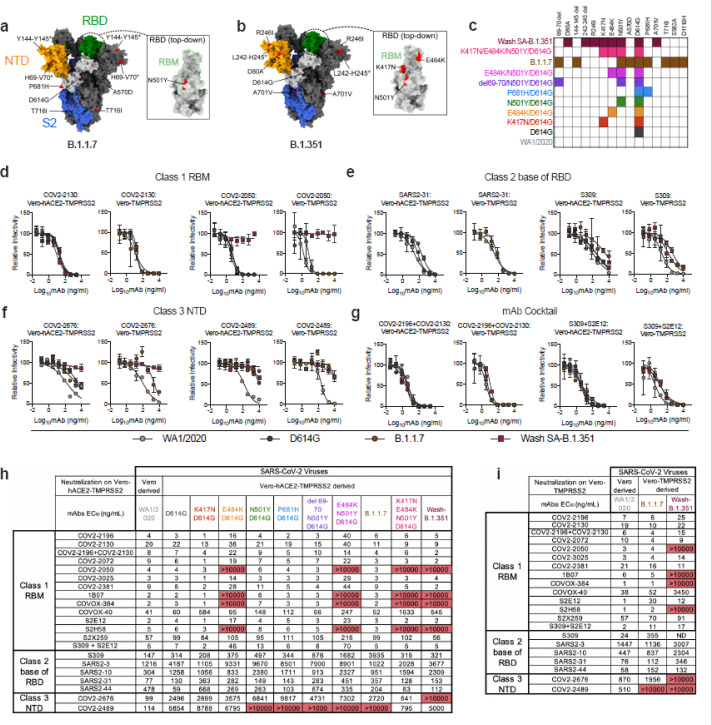
Neutralization of SARS-CoV-2 viral variants by mAbs. a-b, SARS-CoV-2 spike trimer. One protomer is highlighted, showing the NTD in orange, RBD in green, and S2 portion of the molecule in blue, with N- and C-termini annotated. a, Substitutions in the B.1.1.7 variant (69–70 deletion, 144–145 deletion, A570D, D614G, P681H, and T716I) are shaded in red. Red triangle depicts approximate location of P681H, which was not included in the model. Inset shows a top-down view of the RBD showing the location of the N501Y mutation contextualized with the receptor-binding motif (RBM). b, Substitutions in the Wash-SA B.1.135 variant (242–244 deletion, D80A, R246I, D614G, and A701V) are shaded in red. The red diamond denotes approximate location of D80A, which is buried in this view. Inset shows top-down view of the RBD with Wash SA-B.1.351 substitutions K417N, E484K, and N501Y shaded red and contextualized with the receptor binding motif. For all panels, structures depicting spike protein were modeled using PDB: 7C2L. Structures depicting RBD were modeled using PDB: 6W41. All analyses and figures were generated with UCSF ChimeraX40. c, Viruses with indicated spike mutations used in this study. d-f, Neutralization curves in (left panels) Vero-hACE2-TMPRSS2 cells or (right panels) Vero-TMPRSS2 cells comparing the sensitivity of SARS-CoV-2 strains with class 1 (d, COV2–2130 and COV-2150), class 2 (e, SARS2–31 and S309), and class 3 (f, COV2–2676 and COV2–2489) mAbs and indicated viruses. Also shown are the neutralization curves for antibody cocktails (g, COV2–2196 + COV2–2130 and S309 + S2E12). One representative experiment of two is shown. h-i, Summary of EC50 values (ng/ml) of neutralization of SARS-CoV-2 viruses propagated on the indicated cells and performed in Vero-hACE2-TMPRSS2 (h) or Vero-TMPRSS2 (i) cells. Data are an average of two experiments, each performed in duplicate. Red shading of cells shows virtually complete loss of neutralizing activity: EC50 > 10,000 ng/mL.

**Figure 2:**
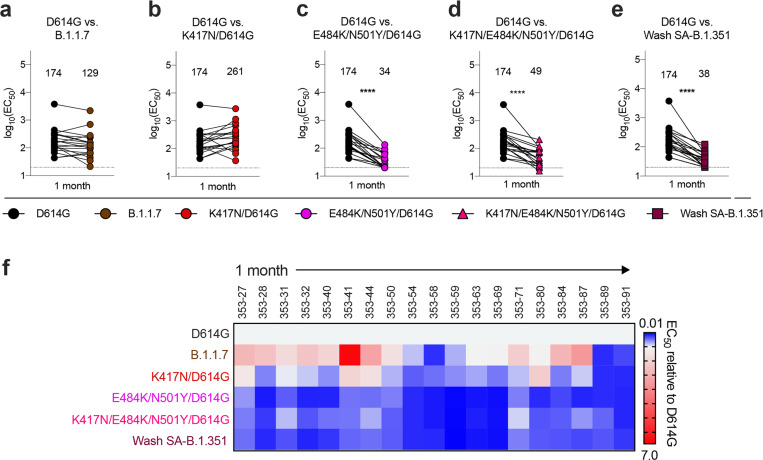
Neutralization of SARS-CoV-2 viral variants by convalescent human serum in Vero-hACE2 TMPRSS2 cells. a-e, Paired neutralization analysis of convalescent human sera (n = 19) obtained approximately 1 month after mild SARS-CoV-2 infection against WA1/2020 D614G and variant viruses in Vero-hACE2-TMPRSS2 cells: (a) B.1.1.7, (b) K417N/D614G, (c) E484K/N501Y/D614G, (d) K417N/E484K/N501Y/D614G, or (e) Wash SA-B.1.351. Results are from one experiment performed in duplicate (Wilcoxon matched-pairs signed rank test, **, P < 0.01; ****, P < 0.0001; all other comparisons, not significant). Geometric mean neutralization titers (GMT) are shown above each graph. Dotted line represents the limit of detection of the assay. f, Heat map of the relative neutralizing activity of sera from individual convalescent subjects against indicated SARS-CoV-2 viruses compared to recombinant WA1/2020 D614G. Blue, reduction; red, increase.

**Figure 3:**
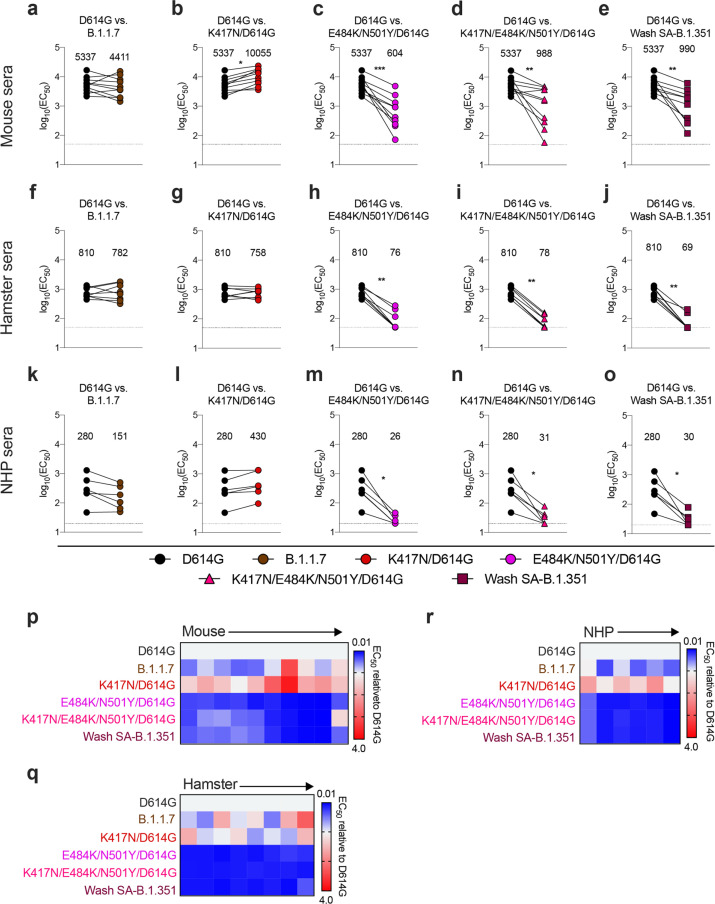
Resistance of SARS-CoV-2 viral variants to neutralization by vaccine-induced serum derived from mice, hamsters, and NHPs. Paired neutralization analysis of sera from mice (a-e, n = 10), hamsters (f-j, n = 8), and NHPs (k-o, n = 6) obtained ~30 days after a single intranasal immunization with an adenoviral vectored SARS-CoV-2 vaccine (ChAd-SARS-CoV-2-S21). Neutralization data on Vero-hACE2-TMPRSS2 cells is displayed as WA1/2020 D614G versus the variant viruses: (a, f, k) B.1.1.7, (b, g, l) K417N/D614G, (c, h, m) E484K/N501Y/D614G, (d, i, n) K417N/E484K/N501Y/D614G, or (e, j, o) Wash SA-B.1.351. EC50 values were calculated from one experiment each performed in duplicate with some exceptions due to limited sample (Wilcoxon matched-pairs signed rank test, *, P < 0.05; **, P < 0.01; ***, P < 0.001; all other comparisons, not significant). GMT values are shown above each graph. Dotted line represents the limit of detection of the assay. p-r, Heat map of the relative neutralizing activity of sera from individual mice (p), hamsters (q), and NHPs (r) against indicated SARS-CoV-2 viruses compared to WA1/2020 D614G. Blue, reduction; red, increase.

**Figure 4:**
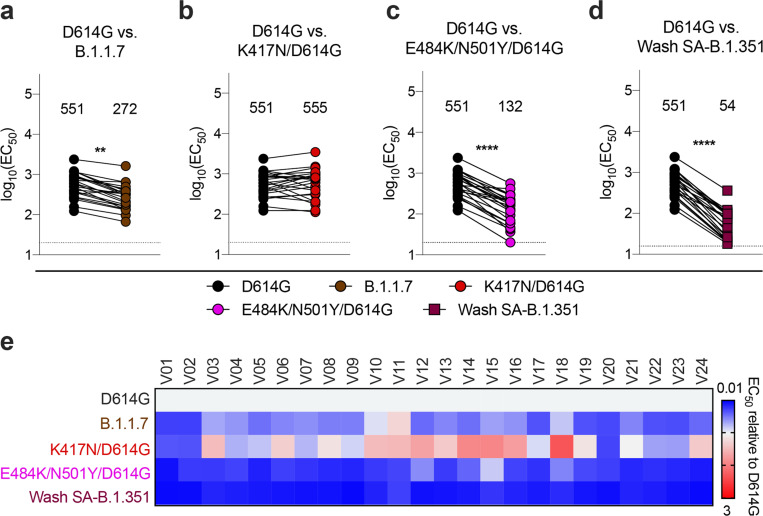
Resistance of SARS-CoV-2 viral variants to neutralization by human serum from Pfizer-BioNTech BNT162b2 mRNA vaccinated individuals in Vero-hACE2-TMPRSS2 cells. Paired neutralization analysis of sera from humans (n = 24) obtained after boosting with the BNT162b2 mRNA vaccine. Neutralization data on Vero-hACE2-TMPRSS2 cells is displayed with WA1/2020 D614G versus the variant viruses: (a) B.1.1.7, (b) K417N/D614G, (c) E484K/N501Y/D614G, or (d) Wash SA-B.1.351. EC50 values were calculated from one experiment each performed in duplicate (Wilcoxon matched-pairs signed rank test, **, P < 0.01; ****, P < 0.0001; all other comparisons, not significant). GMT values are shown above each graph. Dotted line represents the limit of detection of the assay. e, Heat map of the relative neutralizing activity of sera from vaccinated individuals against indicated SARS-CoV-2 viruses compared to WA1/2020 D614G. Blue, reduction; red, increase.

**Figure 5:**
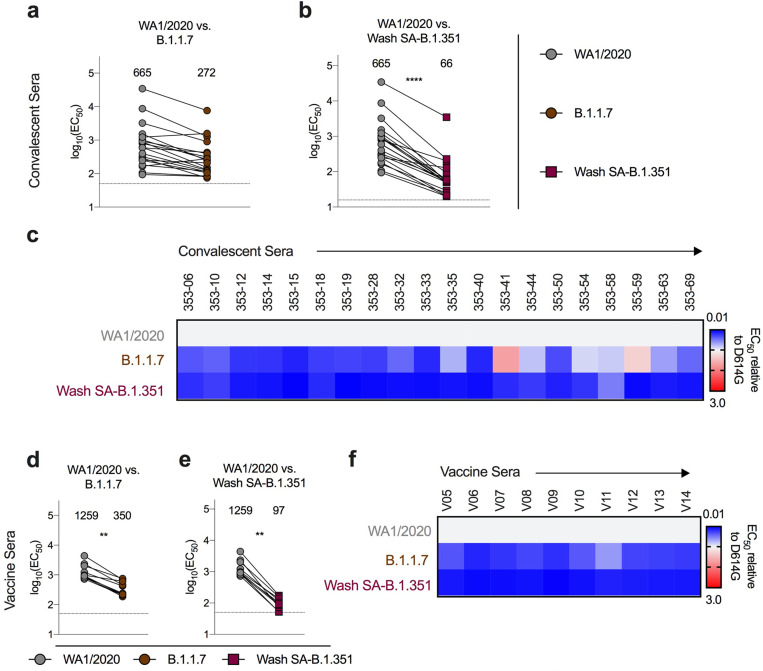
Resistance of SARS-CoV-2 viral variants to neutralization by human serum from convalescent and vaccinated individuals in Vero-TMPRSS2 cells. Sera from individuals who had been infected with SARS-CoV-2 (a-c; n = 20, ~1-month post-infection) or vaccinated with the Pfizer-BioNTech mRNA vaccine (d-f; n = 10) were tested for neutralization of the indicated SARS-CoV-2 strains (WA1/2020 (a, b, d, e), B.1.1.7 (a, d), or Wash SA-B.1.351 (b, e) using a FRNT in Vero-TMPRSS2 cells. EC50 values were calculated from one experiment performed in duplicate (Wilcoxon matched-pairs signed rank test, **, P < 0.01). GMT values are shown above each graph. Dotted line represents the limit of detection of the assay. c, f, Heat maps of the relative neutralizing activity of sera from convalescent (c) or vaccinated (f) individuals against indicated SARS-CoV-2 viruses compared to WA1/2020. Blue, reduction; red, increase.

**Table 1.** Monoclonal antibodies used in this study

	MAb	Region	Species	EC50 value (ng/ml) ^a^	block ACE2 binding	Binds RBM	Structural contacts	Functionally important residues ^b^	Other mapping data	Reference
**CLASS 1**	COV2-2196	RBD - RBM	human	15	yes	yes		F486A, N487A (LOB)		(Dong et al., 2021; Zost et al., 2020a; Zost et al., 2020b)
	COV2-2130	RBD - RBM	human	107	yes	yes		K444A, G447R (LOB); and R346I, K444R, K444E (NE)		(Dong et al., 2021; Zost et al., 2020a; Zost et al., 2020b)
	COV2-2072	RBD - RBM	human	26	yes	yes			COV2-2196 competitive	(Zost et al., 2020a; Zost et al., 2020b)
	COV2-2050	RBD - RBM	human	80	yes	yes		E484K (LOB and NE)	COV2-2196 and COV2-2130 competitive	(Greaney et al., 2021; Zost et al., 2020a; Zost et al., 2020b)
	COV2-3025	RBD - RBM	human	37	yes	yes			COV2-2196 competitive	(Zost et al., 2020a; Zost et al., 2020b)
	COV2-2381	RBD - RBM	human	42	yes	yes			COV2-2196 competitive	(Zost et al., 2020a; Zost et al., 2020b)
	1B07	RBD - RBM	mouse-human chimera	279	yes	yes		E484A/D/G/K, F486Y (NE)		(Alsoussi et al., 2020; Liu et al., 2020)
	S2E12	RBD - RBM	human	4.2	yes	yes	455 to 458, 473 to 493	G476S, F486A (LOB)		(Tortorici et al., 2020), Starr, Corti et al. unpublished
	COVOX-384	RBD - RBM	human	2	yes	yes	L455, F456, G482-F486			Dejnirattisai and Screaton, unpublished
	COVOX-40	RBD - RBM	human	24	yes	yes	K417, Q409, Y505			Dejnirattisai and Screaton, unpublished
	S2H58	RBD-RBM	human	5	yes	yes		E484K, F490L, S494P (LOB)		Starr, Corti et al. unpublished
	S2X259	RBD	human	55	yes	no		G504D (LOB)		McCallum, Corti et al, unpublished
**CLASS 2**	S309	RBD-BASE	human	79	no	no	T333-L335,P337,G339-V341,N343R346,N354,K356-C361,N440L441,K444,R509			(Pinto et al., 2020)
	SARS2-31	RBD-BASE	mouse	246	yes	no		K378E/Q, R408K, G504D (NE)	CR3022 competitive	VanBlargan and Diamond, unpublished
	SARS2-10	RBD-BASE	mouse	694	yes	no			CR3022 competitive	VanBlargan and Diamond, unpublished
	SARS2-54	RBD-BASE	mouse	32	yes	no			CR3022 competitive	VanBlargan and Diamond, unpublished
	SARS2-44	RBD-BASE	mouse	258	yes	no			CR3022 competitive	VanBlargan and Diamond, unpublished
	SARS2-3	RBD-BASE	mouse	670	no	no			CR3022 competitive	VanBlargan and Diamond, unpublished
	SARS2-65	RBD-BASE	mouse	145	no	no			CR3022 competitive	VanBlargan and Diamond, unpublished
**CLASS 3**	COV2-2676	NTD	human	501	no	no		Y144A, N164A (LOB) and F140S (NE)		(Suryadevara et al., 2021; Zost et al., 2020a; Zost et al., 2020b)
	COV2-2489	NTD	human	199	no	no		G142A, Y144A, F157A, N164A (LOB); and G142D, R158S (NE)		(Suryadevara et al., 2021; Zost et al., 2020a; Zost et al., 2020b)

a Neutralization potency determined by FRNT assay with WA1/2020 isolate.

b LOB, loss-of-binding to spike protein, and NE, neutralization escape mutants.

## References

[1] Sempowski G. D., Saunders K. O., Acharya P., Wiehe K. J. & Haynes B. F. Pandemic Preparedness: Developing Vaccines and Therapeutic Antibodies For COVID-19. Cell 181, 1458–1463, doi:10.1016/j.cell.2020.05.041 (2020). 32492407 PMC7250787

[2] Wibmer C. K. SARS-CoV-2 501Y.V2 escapes neutralization by South African COVID-19 donor plasma. bioRxiv, doi:10.1101/2021.01.18.427166 (2021). 33654292

[3] Wang Z. mRNA vaccine-elicited antibodies to SARS-CoV-2 and circulating variants. bioRxiv, doi:10.1101/2021.01.15.426911 (2021). PMC8503938 33567448

[4] Wang P. Increased Resistance of SARS-CoV-2 Variants B.1.351 and B.1.1.7 to Antibody Neutralization. bioRxiv, doi:10.1101/2021.01.25.428137 (2021).

[5] Leung K., Shum M. H., Leung G. M., Lam T. T. & Wu J. T. Early transmissibility assessment of the N501Y mutant strains of SARS-CoV-2 in the United Kingdom, October to November 2020. Euro Surveill 26, doi:10.2807/1560-7917.es.2020.26.1.2002106 (2021). PMC7791602 33413740

[6] Klimstra W. B. SARS-CoV-2 growth, furin-cleavage-site adaptation and neutralization using serum from acutely infected hospitalized COVID-19 patients. J Gen Virol 101, 1156–1169, doi:10.1099/jgv.0.001481 (2020). 32821033 PMC7879561

[7] Johnson B. A. Loss of furin cleavage site attenuates SARS-CoV-2 pathogenesis. Nature, doi:10.1038/s41586-021-03237-4 (2021). PMC8175039 33494095

[8] Rappazzo C. G. Broad and potent activity against SARS-like viruses by an engineered human monoclonal antibody. Science, doi:10.1126/science.abf4830 (2021). PMC7963221 33495307

[9] Case J. B. Neutralizing antibody and soluble ACE2 inhibition of a replication-competent VSV-SARS-CoV-2 and a clinical isolate of SARS-CoV-2. Cell Host and Microbe 28, 475–485 (2020). 32735849 10.1016/j.chom.2020.06.021 PMC7332453

[10] Zost S. J. Rapid isolation and profiling of a diverse panel of human monoclonal antibodies targeting the SARS-CoV-2 spike protein. Nat Med 26, 1422–1427, doi:10.1038/s41591-020-0998-x (2020). 32651581 PMC8194108

[11] Zost S. J. Potently neutralizing and protective human antibodies against SARS-CoV-2. Nature 584, 443–449, doi:10.1038/s41586-020-2548-6 (2020). 32668443 PMC7584396

[12] Alsoussi W. B. A Potently Neutralizing Antibody Protects Mice against SARS-CoV-2 Infection. J Immunol, doi:10.4049/jimmunol.2000583 (2020). PMC7566074 32591393

[13] Tortorici M. A. Ultrapotent human antibodies protect against SARS-CoV-2 challenge via multiple mechanisms. Science 370, 950–957, doi:10.1126/science.abe3354 (2020). 32972994 PMC7857395

[14] Pinto D. Cross-neutralization of SARS-CoV-2 by a human monoclonal SARS-CoV antibody. Nature 583, 290–295, doi:10.1038/s41586-020-2349-y (2020). 32422645

[15] Suryadevara N. Neutralizing and protective human monoclonal antibodies recognizing the N-terminal domain of the SARS-CoV-2 spike protein. bioRxiv, doi:10.1101/2021.01.19.427324 (2021). PMC7962591 33773105

[16] Xie X. Neutralization of SARS-CoV-2 spike 69/70 deletion, E484K and N501Y variants by BNT162b2 vaccine-elicited sera. Nature Medicine, In press (2021). 10.1038/s41591-021-01270-4 33558724

[17] Wu K. mRNA-1273 vaccine induces neutralizing antibodies against spike mutants from global SARS-CoV-2 variants. bioRxiv, doi:10.1101/2021.01.25.427948 (2021).

[18] Rathnasinghe R. The N501Y mutation in SARS-CoV-2 spike leads to morbidity in obese and aged mice and is neutralized by convalescent and post-vaccination human sera. medRxiv : the preprint server for health sciences, doi:10.1101/2021.01.19.21249592 (2021).

[19] Baum A. Antibody cocktail to SARS-CoV-2 spike protein prevents rapid mutational escape seen with individual antibodies. Science, doi:10.1126/science.abd0831 (2020). PMC7299283 32540904

[20] Ellebedy A. SARS-CoV-2 infection induces long-lived bone marrow plasma cells in humans. Research square, doi:10.21203/rs.3.rs-132821/v1 (2020). 34030176

[21] Hassan A. O. A Single-Dose Intranasal ChAd Vaccine Protects Upper and Lower Respiratory Tracts against SARS-CoV-2. Cell 183, 169–184.e113, doi:10.1016/j.cell.2020.08.026 (2020). 32931734 PMC7437481

[22] Bricker T. L. A single intranasal or intramuscular immunization with chimpanzee adenovirus vectored SARS-CoV-2 vaccine protects against pneumonia in hamsters. bioRxiv, doi:10.1101/2020.12.02.408823 (2020). PMC8238649 34245672

[23] Hassan A. O. A single intranasal dose of chimpanzee adenovirus-vectored vaccine protects against SARS-CoV-2 infection in rhesus macaques. bioRxiv, doi:10.1101/2021.01.26.428251 (2021). PMC7969912 33754147

[24] Polack F. P. Safety and Efficacy of the BNT162b2 mRNA Covid-19 Vaccine. N Engl J Med 383, 2603–2615, doi:10.1056/NEJMoa2034577 (2020). 33301246 PMC7745181

[25] McCallum M. N-terminal domain antigenic mapping reveals a site of vulnerability for SARS-CoV-2. bioRxiv, doi:10.1101/2021.01.14.426475 (2021). PMC7962585 33761326

[26] Greaney A. J. Complete Mapping of Mutations to the SARS-CoV-2 Spike Receptor-Binding Domain that Escape Antibody Recognition. Cell Host Microbe 29, 44–57.e49, doi:10.1016/j.chom.2020.11.007 (2021). 33259788 PMC7676316

[27] Piccoli L. Mapping Neutralizing and Immunodominant Sites on the SARS-CoV-2 Spike Receptor-Binding Domain by Structure-Guided High-Resolution Serology. Cell 183, 1024–1042.e1021, doi:10.1016/j.cell.2020.09.037 (2020). 32991844 PMC7494283

[28] Liu Z. Landscape analysis of escape variants identifies SARS-CoV-2 spike mutations that attenuate monoclonal and serum antibody neutralization. bioRxiv, doi:10.1101/2020.11.06.372037 (2020). PMC7839837 33535027

[29] Weisblum Y. Escape from neutralizing antibodies by SARS-CoV-2 spike protein variants. Elife 9, doi:10.7554/eLife.61312 (2020). PMC7723407 33112236

[30] Liu Z. Identification of SARS-CoV-2 spike mutations that attenuate monoclonal and serum antibody neutralization. Cell Host Microbe, doi:10.1016/j.chom.2021.01.014 (2021). PMC7839837 33535027

[31] Andreano E. SARS-CoV-2 escape in vitro from a highly neutralizing COVID-19 convalescent plasma. bioRxiv, doi:10.1101/2020.12.28.424451 (2020). PMC8433494 34417349

[32] Kim J. H., Marks F. & Clemens J. D. Looking beyond COVID-19 vaccine phase 3 trials. Nat Med, doi:10.1038/s41591-021-01230-y (2021). 33469205

[33] Schäfer A. Antibody potency, effector function, and combinations in protection and therapy for SARS-CoV-2 infection in vivo. J Exp Med 218, doi:10.1084/jem.20201993 (2021). PMC7673958 33211088

[34] Winkler E. S. Human neutralizing antibodies against SARS-CoV-2 require intact Fc effector functions and monocytes for optimal therapeutic protection. bioRxiv, doi:10.1101/2020.12.28.424554 (2020). PMC7879018 33691139

[35] Zohar T. Compromised Humoral Functional Evolution Tracks with SARS-CoV-2 Mortality. Cell 183, 1508–1519.e1512, doi:10.1016/j.cell.2020.10.052 (2020). 33207184 PMC7608014

[36] Dan J. M. Immunological memory to SARS-CoV-2 assessed for up to 8 months after infection. Science, doi:10.1126/science.abf4063 (2021). PMC7919858 33408181

[37] Lipsitch M., Grad Y. H., Sette A. & Crotty S. Cross-reactive memory T cells and herd immunity to SARS-CoV-2. Nat Rev Immunol 20, 709–713, doi:10.1038/s41577-020-00460-4 (2020). 33024281 PMC7537578

[38] Sette A. & Crotty S. Adaptive immunity to SARS-CoV-2 and COVID-19. Cell, doi:10.1016/j.cell.2021.01.007 (2021). PMC7803150 33497610

[39] Bailey A. L. & Diamond M. S. A Crisp(r) New Perspective on SARS-CoV-2 Biology. Cell 184, 15–17, doi:10.1016/j.cell.2020.12.003 (2021). 33338422 PMC7746090

[40] Goddard T. D. UCSF ChimeraX: Meeting modern challenges in visualization and analysis. Protein Sci 27, 14–25, doi:10.1002/pro.3235 (2018). 28710774 PMC5734306

[41] Plante J. A. Spike mutation D614G alters SARS-CoV-2 fitness. Nature, doi:10.1038/s41586-020-2895-3 (2020). PMC8158177 33106671

[42] Xie X. An Infectious cDNA Clone of SARS-CoV-2. Cell Host Microbe 27, 841–848.e843, doi:10.1016/j.chom.2020.04.004 (2020). 32289263 PMC7153529

[43] Xie X. Engineering SARS-CoV-2 using a reverse genetic system. Nat Protoc, doi:10.1038/s41596-021-00491-8 (2021). PMC8168523 33514944

[44] Chen S., Zhou Y., Chen Y. & Gu J. fastp: an ultra-fast all-in-one FASTQ preprocessor. Bioinformatics 34, i884–i890, doi:10.1093/bioinformatics/bty560 (2018). 30423086 PMC6129281

[45] Li H. & Durbin R. Fast and accurate short read alignment with Burrows-Wheeler transform. Bioinformatics 25, 1754–1760, doi:10.1093/bioinformatics/btp324 (2009). 19451168 PMC2705234

[46] Poplin R. A universal SNP and small-indel variant caller using deep neural networks. Nat Biotechnol 36, 983–987, doi:10.1038/nbt.4235 (2018). 30247488

[47] Cingolani P. A program for annotating and predicting the effects of single nucleotide polymorphisms, SnpEff: SNPs in the genome of Drosophila melanogaster strain w1118; iso-2; iso-3. Fly 6, 80–92, doi:10.4161/fly.19695 (2012). 22728672 PMC3679285

