# Supplementary figures and images for: SARS-CoV-2 variants show resistance to neutralization by many monoclonal and serum-derived polyclonal antibodies
Source: Res Sq. 2021 Feb 10:rs.3.rs-228079. Preprint. [Version 1] doi: 10.21203/rs.3.rs-228079/v1 (PMC7885928; doi:10.21203/rs.3.rs-228079/v1)

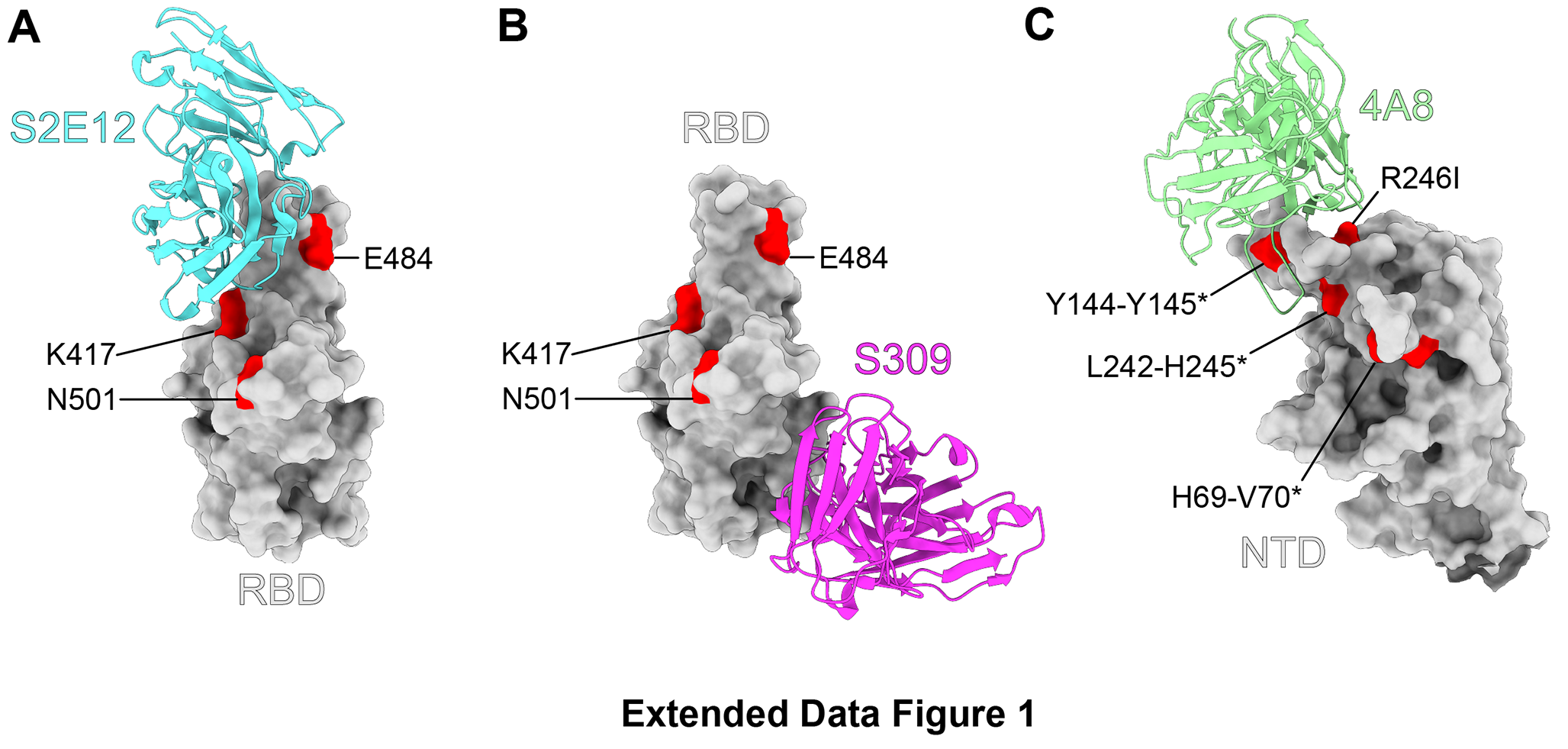

Supplement: Supplement [file 0ce31165bdc570123f6b7d7f.tif]

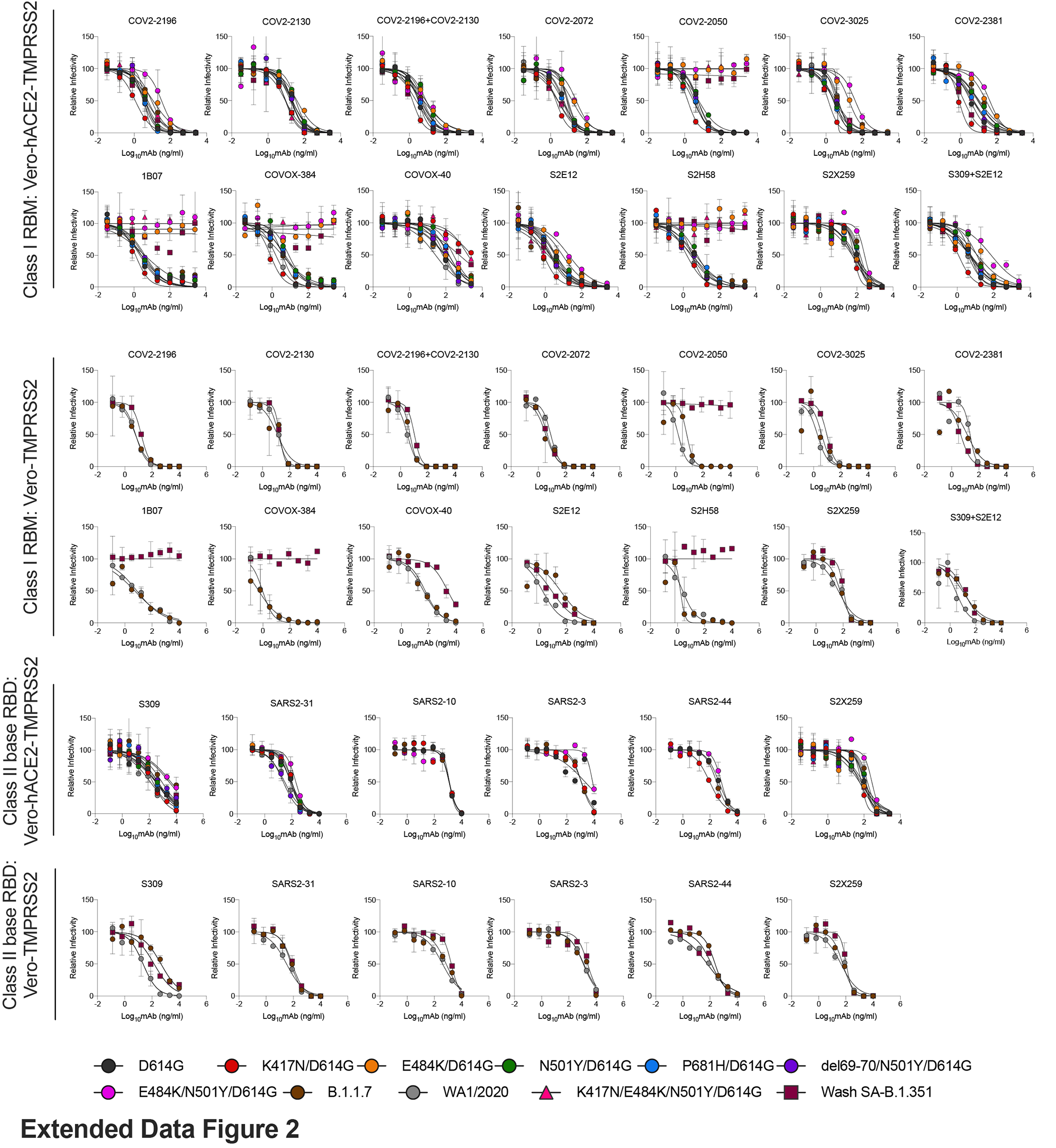

Supplement: Supplement [file 00fe304d4d98db250de472e8.tif]

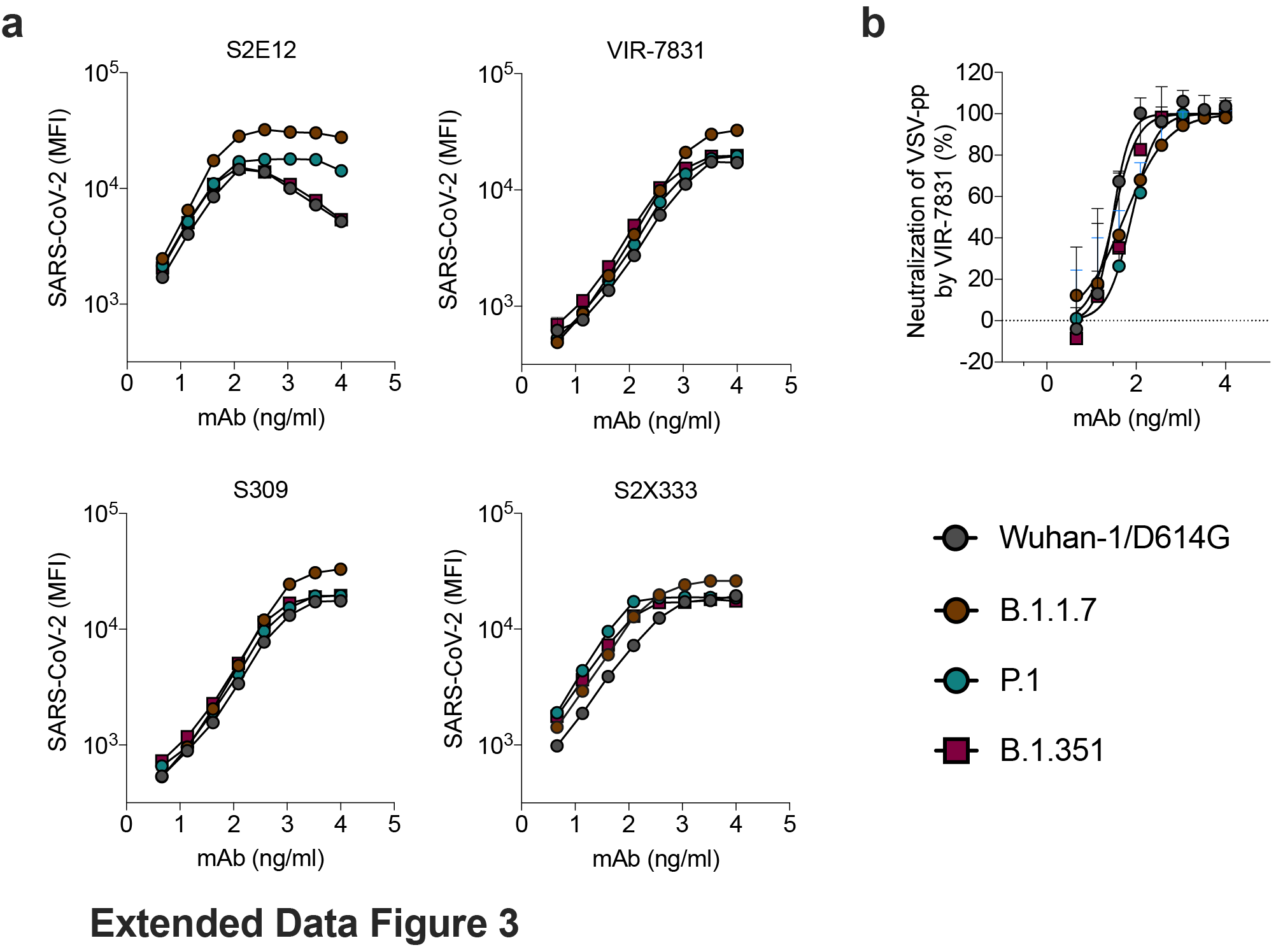

Supplement: Supplement [file 12d9a6bd62819a43582106cf.tif]

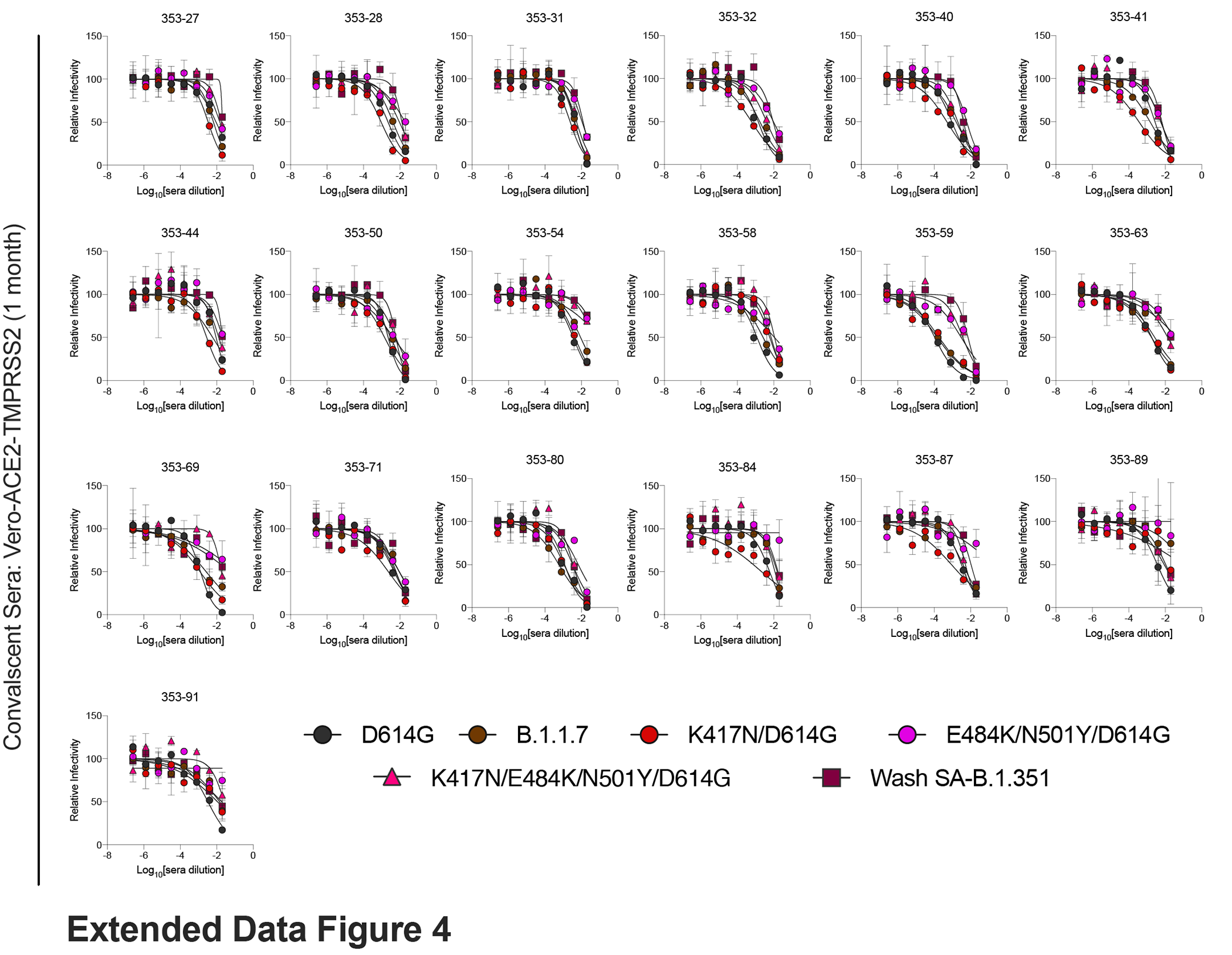

Supplement: Supplement [file e4f2abbe423f7ffb25a879db.tif]

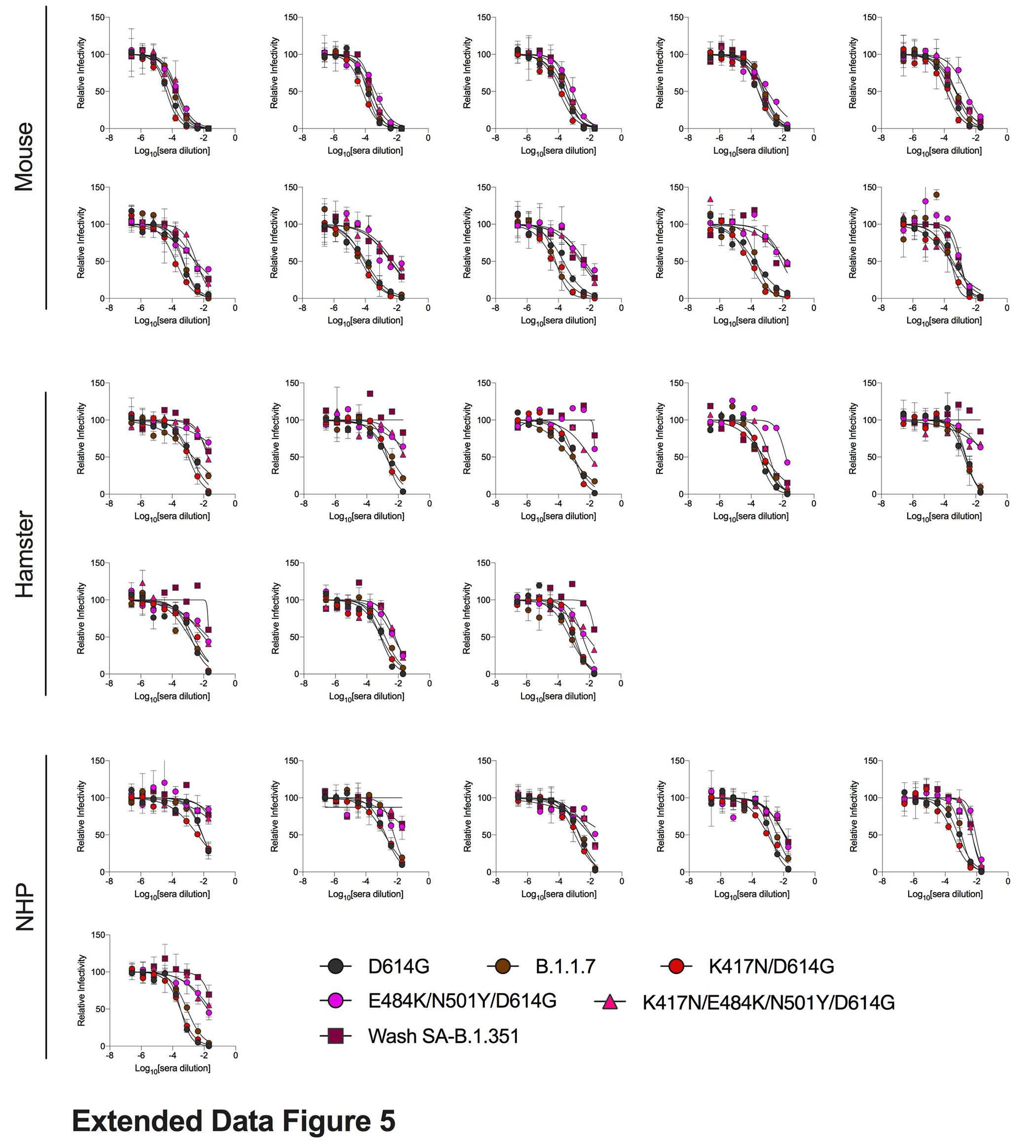

Supplement: Supplement [file 87a46386ae1cb43fb5796df9.tif]

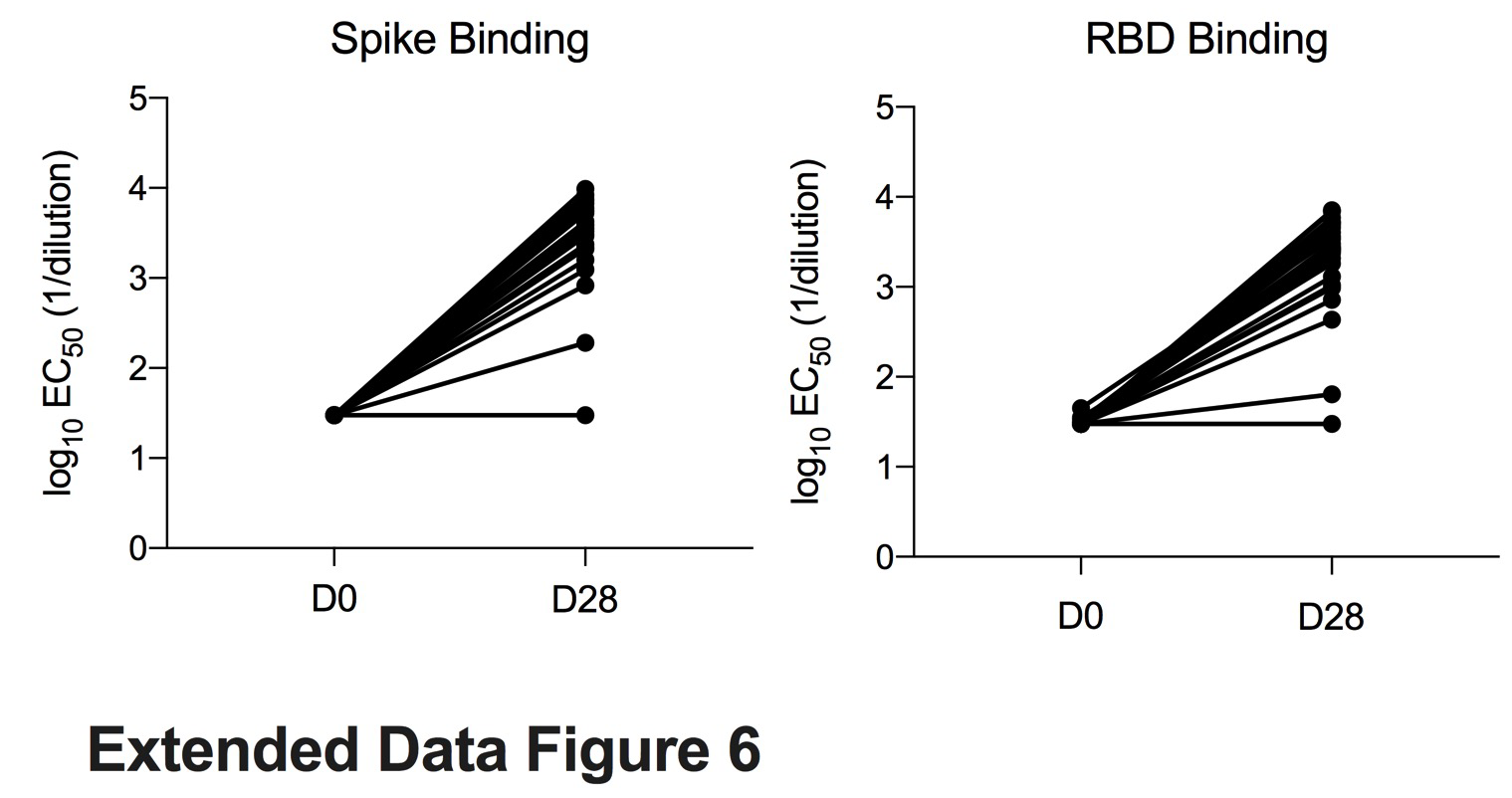

Supplement: Supplement [file 87dc16f24ec95a353bad3551.tif]

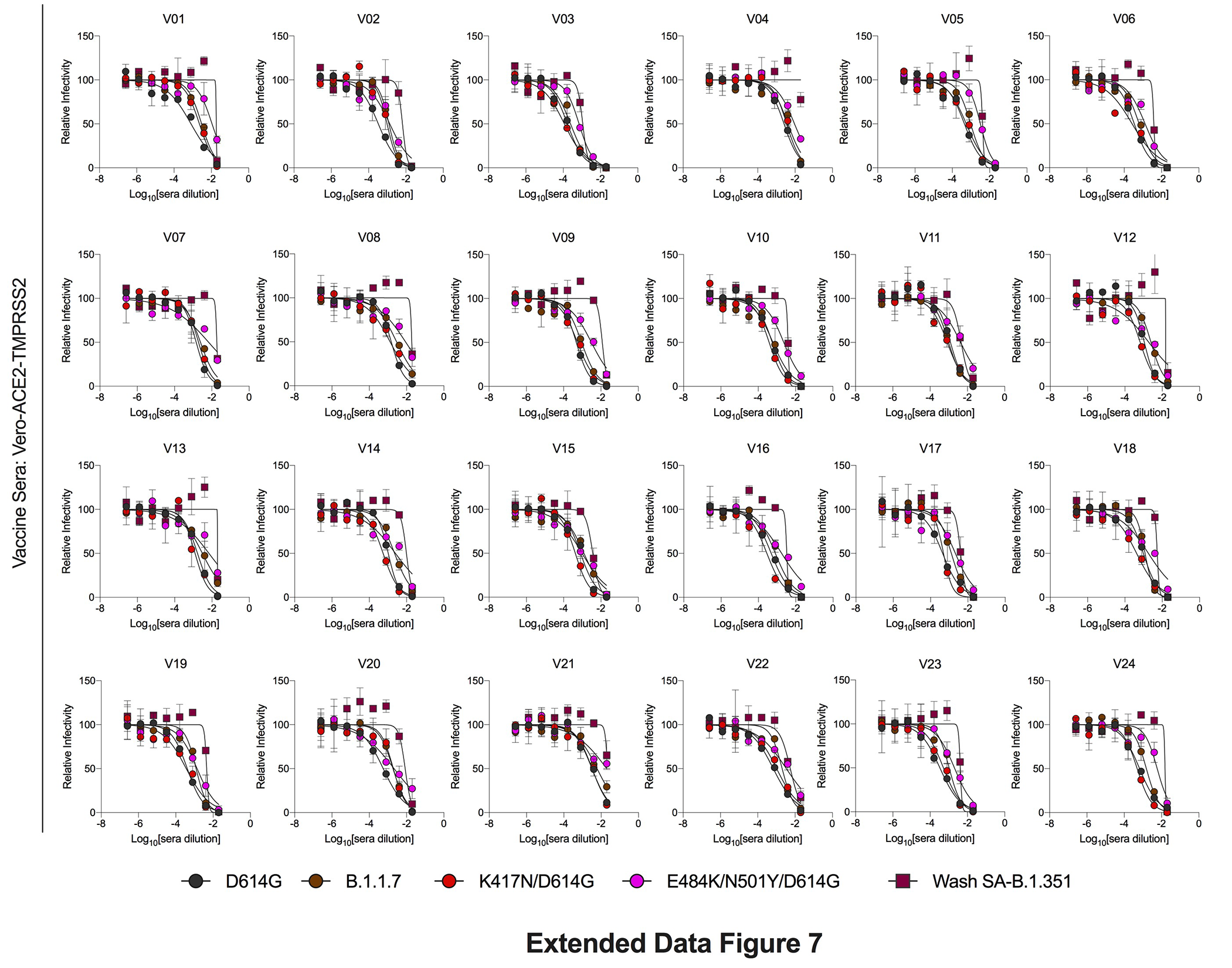

Supplement: Supplement [file 1ad63baba5ab27adf4222bc7.tif]

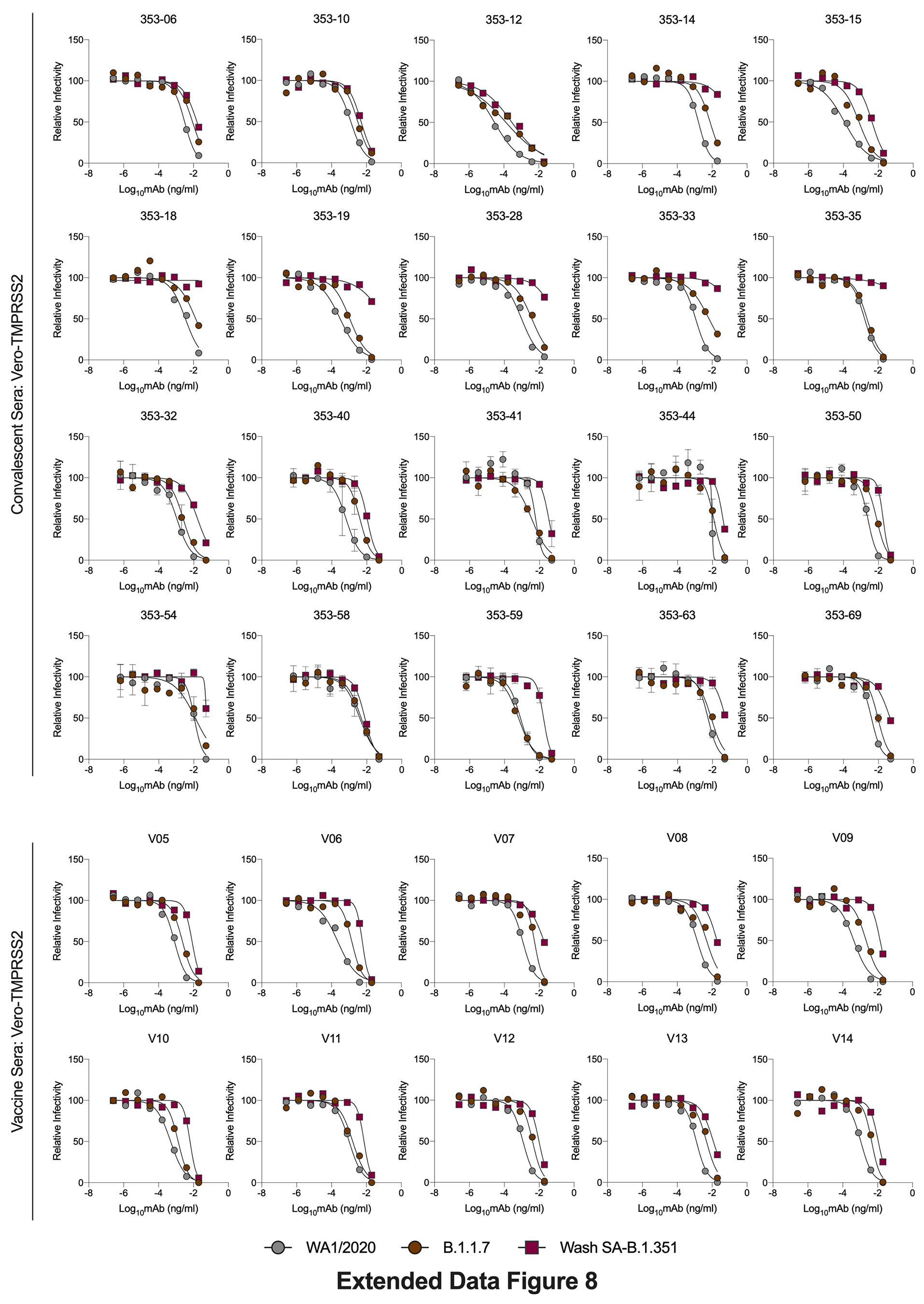

Supplement: Supplement [file e42e197a8496d4465dbd5059.tif]

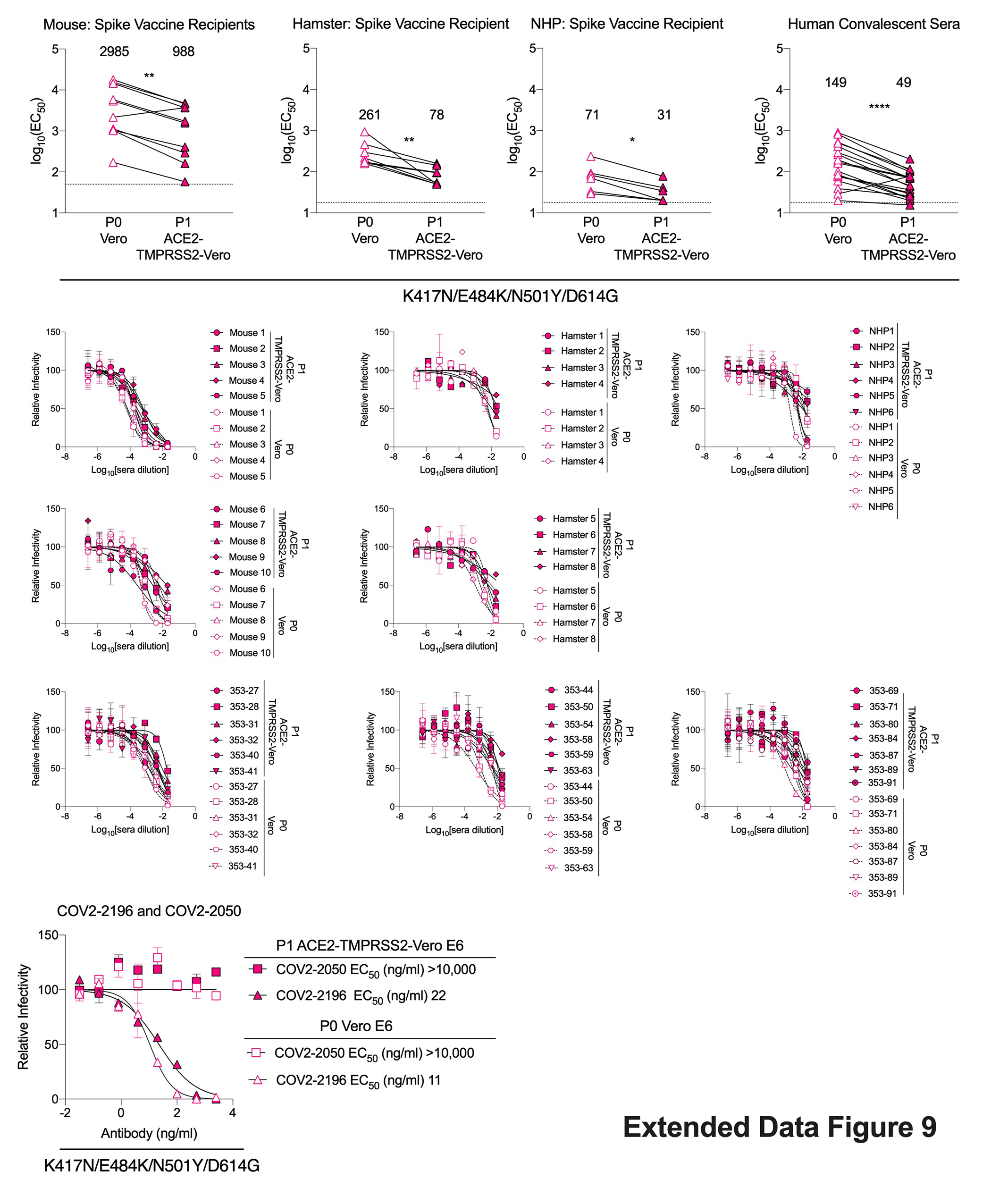

Supplement: Supplement [file 8057cf4216d7b46f89d6e34d.tif]
